# Complexation of 5-Fluorouracil with *β*-Cyclodextrin and Sodium Dodecyl Sulfate: A Useful Tool for Encapsulating and Removing This Polluting Drug

**DOI:** 10.3390/toxics10060300

**Published:** 2022-06-01

**Authors:** Ana M. T. D. P. V. Cabral, Ana C. G. Fernandes, Neuza A. M. Joaquim, Francisco Veiga, Sara P. C. Sofio, Isabel Paiva, Miguel A. Esteso, M. Melia Rodrigo, Artur J. M. Valente, Ana C. F. Ribeiro

**Affiliations:** 1 Faculty of Pharmacy, University of Coimbra, 3000-548 Coimbra, Portugal; acabral@ff.uc.pt (A.M.T.D.P.V.C.); alexandrareix@gmail.com (N.A.M.J.); fveiga@ci.uc.pt (F.V.); 2 Department of Chemistry, CQC, Institute of Molecular Sciences, University of Coimbra, 3004-535 Coimbra, Portugal; anacatarina2813@gmail.com (A.C.G.F.); sarapcsofio@gmail.com (S.P.C.S.); avalente@ci.uc.pt (A.J.M.V.); 3 Centre of Geography and Spatial Planning, Department of Geography and Tourism, University of Coimbra, 3004-530 Coimbra, Portugal; isabelrp@fl.uc.pt; 4 Universidad Católica de Ávila, Calle los Canteros s/n, 05005 Ávila, Spain; 5 U.D. Química Física, Universidad de Alcalá, 28805 Alcalá de Henares, Spain; mmelia.rodrigo@uah.es

**Keywords:** 5-fluorouracil, *β*-cyclodextrin, sodium dodecyl sulphate, complexation, diffusion coefficients

## Abstract

The formation of complexes of the drug 5-fluorouracil (5-FU) with *β*-cyclodextrin (*β*-CD) and sodium dodecyl sulphate (SDS) was studied through experimental measurements of the ternary mutual diffusion coefficients (*D*_11_, *D*_22_, *D*_12_, and *D*_21_) for the systems {5-FU (component 1) + *β*-CD (component 2) + water} and {5-FU (component 1) + SDS (component 2) + water} at 298.15 K and at concentrations up to 0.05 mol dm^−3^ by using the Taylor dispersion method, with the objective of removing this polluting drug from the residual systems in which it was present. The results found showed that a coupled diffusion of 5-FU occurred with both *β*-CD and SDS, as indicated by the nonzero values of the cross-diffusion coefficients, *D*_12_ and *D*_21_, as a consequence of the complex formation between 5-FU and the *β*-CD or SDS species. That is, 5-FU was solubilized (encapsulated) by both carriers, although to a greater extent with SDS (K = 20.0 (±0.5) mol^−1^ dm^3^) than with *β*-CD (K = 10.0 (±0.5) mol^−1^ dm^3^). Values of 0.107 and 0.190 were determined for the maximum fraction of 5-FU solubilized with *β*-CD and SDS (at concentrations above its CMC), respectively. This meant that SDS was more efficient at encapsulating and thus removing the 5-FU drug.

## 1. Introduction

5-fluorouracil, or 5-FU (5-fluoro-1H-pyrimidine-2,4-dione, C_4_H_3_FN_2_O_2_; M = 130.077 g/mol; CAS: 51-21-8), is one of the most relevant anticancer agents that belongs to the family of antimetabolites, and one of the most widely used [1] in the treatment of a wide variety of cancers (its systemic use is indicated in the treatment of adenocarcinomas of the gastrointestinal tract [2,3], lungs, breast, pancreas, ovaries, and prostate [3,4,5]). Moreover, it is also used topically, in emulsion and cream forms, for various dermatological affections (warts, psoriasis, vitiligo [6,7,8], keratoacanthoma, and melanoma [9]).

5-FU is a nonspecific cytostatic agent that, depending on the type of carcinoma to be treated, is administered orally (with an undetermined low efficiency [2,3]), intravenously, and intraperitoneally [3]. In all these cases, due to its low bioavailability [10], the doses used are very high and its utilization is small, so that a significant part of the administered drug (approximately 35% [11]) is excreted by the patient through urine [12] and subsequently passed into wastewater. In fact, there is an abundance of this 5-FU drug in hospital wastewater. When the drug is administered topically to treat skin lesions, it is also applied in high doses due to its low penetrating power [13,14,15]; the unused surplus then ends up in the wastewater. That is, this drug is one of the polluting agents in environmental contamination by hospital effluents. Various studies have been performed to determine the amounts of several drugs (including 5-FU) in wastewater, as well as their biodegradability, which have yielded quite different results among them. Despite the contradictory results, practically all of them agreed that their presence was abundant. For example, the expected range of contamination in wastewater from the oncology departments of the Vienna University Hospital was 5–500 µg 5-FU L^−1^ [16]. Likewise, a 2009 publication by Corporate Safety, Health & Environmental Protection, Switzerland, reported a figure of 1545 kg of 5-FU per year excreted into wastewater in Europe [17]. The use of carriers (nanoparticles and vesicles) that facilitate an efficient delivery system for this drug helps to improve this situation [18,19,20], although the definitive solution to the problem is still far from being obtained.

Due to its hydrophilic nature, 5-FU is not easily removable in wastewater treatment plants (WWTPs), as it is not adsorbed in the sludge that is moved away in the secondary [21]. This fact, together with the long half-life that 5-FU has in aqueous media (approximately 360 h in water), means that this drug accumulates in aquatic media, and therefore, due to its potential toxicity, even at low concentrations [22,23], it may eventually be capable of causing long-term effects in the environment and wildlife [24], as well as in human health [23]. Based on these facts, it is very important to find effective processes to remove this 5-FU drug from wastewater.

One possible way is to encapsulate it is by using a complexing agent and then removing it. Numerous studies have been devoted to complexes of cyclodextrins [25,26,27] and micelles [28] with different drugs in aqueous solutions, as well as to the possible modification of the molecular structure of these complexing agents to improve the ability to encapsulate different drugs [29].

Likewise, the use of liposomes as carriers/encapsulating agents of 5-FU has been extensively studied [30,31,32] to both improve its bioavailability and reduce side effects, as compared to traditional formulations used under the form of creams and/or emulsions [33,34,35], for topical use in the treatment of different skin diseases [19,36]. Lakkakula et al. [20] found that 5-FU could be encapsulated in the cavity of 2HP-*β*-cyclodextrin, forming a stable inclusion complex, although they did not report the extent of such complex formation.

The present work focused on the study of the possible complexation of the drug 5-FU with *β*-cyclodextrin (*β*-CD) and with sodium dodecyl sulphate (SDS) as a procedure for the extraction/removal of said drug from wastewater. These compounds were chosen while keeping in mind that *β*-CD is a proven complexing agent for several drugs, and therefore is widely used as a carrier in drug delivery systems [27,37]; and that SDS is an anionic surfactant widely used in the biochemical, pharmaceutical, and food industries that possesses striking abilities as a complexing agent [38,39].

The possible formation of such complexes was followed by measuring the diffusion coefficient of 5-FU in aqueous solutions containing either the carrier *β*-cyclodextrin or the carrier sodium dodecyl sulphate. This simple measurement of the diffusion of the species present in the medium allowed us to directly determine the molecular behavior and organization in the solution while also quantitatively evaluating the formation of molecular associations that may originate between 5-FU and the carrier.

## 2. Materials and Methods

### 2.1. Materials

Table 1 presents the reagents used in this work; they were used as received, except after drying, they were stored in a desiccator over silica gel. Solutions were prepared in calibrated volumetric glass flasks using ultrapure water as a solvent (specific resistance = 18.2 MΩ·cm at 298.15 K). Weighing was performed with a Radwag AS 220C2 balance with an accuracy of ±0.0001 g. The water content of the CD (water mass fraction 0.131) was checked by drying to constant mass for 24 h at *T* = 420 K in a nitrogen atmosphere [40], and this value was taken into account to determine the solution concentration. All solutions were freshly prepared at 298.15 K just before each experiment.

### 2.2. Diffusion Measurements: Short Description of the Taylor Dispersion Method

Mutual diffusion coefficients for ternary {5-FU (1) + *β*-CD (2) + water} and {5-FU (1) + SDS (2) + water} solutions are described by Fick’s Equations (1) and (2) [41,42]:

(1) $$J_{1}=-D_{11}\nabla C_{1}-D_{12}\nabla C_{2}$$

(2) $$\;\;J_{2}=-D_{22}\nabla C_{2}-D_{21}\nabla C_{1}$$

*J*_1_ and *J*_2_ are, respectively, the molar fluxes of component (1) (5-FU) and component (2) (*β*-CD or SDS) driven by the concentration gradients of component (1), ∇*C*_1_, and of component (2), ∇*C*_2_. The main diffusion coefficients, *D*_11_ and *D*_22_, give the flux of each component, (1) and (2), respectively, driven by its own concentration gradient. The cross-diffusion coefficients, *D*_12_ and *D*_21_, give the coupled flux of each component, (1) and (2), driven by a concentration gradient in the other component, (2) and (1), respectively. A positive value of the *D*_ik_ cross-coefficient (*i* ≠ *k*) indicates a co-current coupled transport of component (*i*) from regions of higher to lower concentrations of component (*k*). On the other hand, a negative value of *D*_ik_ cross-coefficient (*i* ≠ *k*) indicates a counter-current coupled transport of component (*i*) from regions of lower to higher concentration of component (*k*).

These transport coefficients (*D*_11_, *D*_12_, *D*_21_, and *D*_22_) were determined using the Taylor dispersion technique. Considering that said technique is well described in the literature [41,42,43,44], we will only indicate some relevant characteristics regarding the experimental procedure and the equipment used in this work. At the start of each run, a 0.063 cm^3^ sample of solution was injected into a laminar carrier solution, of slightly different composition, at the entrance of a Teflon capillary dispersion tube (length 3048.0 (±0.1) cm and internal radius 0.03220 (±0.00003) cm). This tube and the injection valve were kept at 298.15 (±0.01) K in an air thermostat. The broadened distribution of the disperse samples was monitored at the tube outlet using a differential refractometer (Waters model 2410). The refractometer output voltages, *V*(*t*), were measured at 5 s intervals using a digital voltmeter (Agilent 34401 A).

Dispersion profiles for ternary mixed solutions {5-FU (1) + *β*-CD (2) (or SDS)} were analyzed by fitting the following equation (Equation (3)):

(3) $$V\left(t\right)=V_{0}+V_{1}t+V_{\max}{\left(\frac{t_{R}}{t}\right)}^{\frac{1}{2}}\left[W_{1}\exp\left(-\frac{12D_{1}{\left(t-t_{R}\right)}^{2}}{r^{2}t}\right)+\left(1-W_{1}\right)\exp\left(-\frac{12D_{2}{\left(t-t_{R}\right)}^{2}}{r^{2}t}\right)\right],$$

where *V*_0_, *V*_1_, and *V*_max_ represent the baseline voltage, the baseline slope, and the peak height relative to the linear baseline voltage *V*_0_ + *V*_1_*t*, respectively; *t_R_* is the mean sample retention time; and *D*_1_ and *D*_2_, are the eigenvalues of the ternary diffusion coefficient matrix.

## 3. Results and Discussion

### 3.1. Ternary Mutual Diffusion Coefficients of Aqueous 5-FU (C_1_) + β-CD (C_2_) Solutions

Table 2 shows the ternary aqueous diffusion for the systems {5-fluorouracil + *β*-cyclodextrin (*β*-CD)} at 298.15 K. The main diffusion coefficients (*D*_11_ and *D*_22_) were generally reproducible within (±0.02 × 10^−9^ m^2^ s^−1^), and the cross-diffusion coefficients (*D*_12_ and *D*_21_) were reproducible within about (±0.05 × 10^−9^ m^2^ s^−1^).

The negative *D*_12_ and *D*_21_ values observed for different 5-FU solute mole fractions, defined as *X*_1_ = *C*_1_/(*C*_1_+*C*_2_), generally indicated that there were counter-current coupled flows; that is, a flux of 5-FU in the opposite direction to the spontaneous flux of *β*-CD, and vice versa.

Furthermore, in the limit X_1_ → 0, *D*_12_ was zero since, due to the fact that the solution does not contain 5-FU, there could be no *β*-CD coupled flows caused by the 5-FU concentration gradient. In those circumstances, *D*_11_ represented the tracer diffusion coefficient of 5-FU in *β*-CD aqueous solutions, and *D*_22_ was the binary mutual diffusion coefficient of *β*-CD. At this concentration, *D*_21_ reached the maximum value.

In the opposite limit (X_1_ → 1), *D*_12_ was different from zero, reaching the maximum value, and *D*_21_ was zero because there was no *β*-CD in the solution and, consequently, no concentration gradient of *β*-CD either, so there could be no 5-FU coupled flows. In this case, *D*_11_ was the binary mutual diffusion coefficient of 5-FU, and *D*_22_ was the tracer diffusion coefficient of *β*-CD in 5-FU aqueous solutions.

Considering that *D*_12_/*D*_22_ gives the number of moles of 5-FU counter-transported per mole of *β*-CD, we can say that, at the concentrations used, a mole of diffusing *β*-CD counter-transported at most 0.2 mol of 5-FU, with the counter-transport increasing with the increase in its concentration. Using *D*_21_/*D*_11_ values at the same concentrations, we could expect that a mole of diffusing 5-FU counter-transported, at most, 0.03 mol of *β*-CD. This observed coupled diffusion in the {5-FU + *β*-CD} solutions could be a consequence of the effects of complex formation of type 1:1 between 5-FU and *β*-CD species (Equation (4)), as supported by NMR data [45]. Considering the model developed by Paduano et al. [46], this equilibrium constant could be estimated. Since this theory is well described in the literature, only the main details are indicated here.

That is, considering the following equilibrium:

5-FU (1) + *β*-CD (2) ⇆ 5-FU-*β*-CD (3) (4)

the association constant, *K*, that describes the stability of these complexes, is given by Equation (5):

(5) $$K=\frac{C_{\left(5\text{-}\mathrm{FU}\text{-}\beta \mathrm{CD}\right)}}{C_{5\text{-}\mathrm{FU}}\;C_{\beta \mathrm{CD}}}$$

where *C*_5-FU_ and *C_β-_*_CD_ represent the concentrations of free 5-FU and *β*-CD, respectively, and *C_(_*_5-FU-*β*-CD)_ is the concentration of the (5-FU-*β*-CD) complex, which are correlated by the following mass balance equations:

*C*_1_ = *C*_5-FU_ + *C_(_*_5-FU-_*_β_*_-__CD)_ (6)

*C*_2_ = *C_β_*_-__CD_ + *C_(_*_5-FU-_*_β_*_-__CD)_ (7)

After labeling these solute species as 5-FU = 1, *β*-CD = 2, and 5-FU-*β*-CD complexes = 3, respectively, Equations (1) and (2) can be rewritten as

*J*_1_ = −*D*_11_∇*C*_1_ − *D*_12_∇*C*_2_ − *D*_13_∇*C*_3_ (8)

*J*_2_ = −*D*_21_∇*C*_1_ − *D*_22_∇*C*_2_ − *D*_23_∇*C*_3_ (9)

Considering that we had diluted solutions, and thus, assuming, as an approach, that the cross-diffusion terms *D*_13_ and *D*_23_ were negligible (i.e., *D*_13_ = *D*_23_ = 0), by inserting this information in the Equations (8) and (9), and after some mathematical rearrangement, it is possible to obtain Equations (10)–(13). These equations provide the relationships between the mutual diffusion coefficients *D*_11_, *D*_12_, *D*_21_, and *D*_22_ measured for the total 5-FU (1) + *β*-CD (2) solute components, and the diffusion coefficients *D*_5-FU_, *D_β_*_-CD_, and *D*_(5-FU-_*_β-_*_CD)_, which represent the diffusion coefficients of the free 5-FU, the free *β*-CD, and the corresponding complex, respectively.

(10) $$D_{11}=\frac{1}{2}\left\{\left(D_{5\text{-}\mathrm{FU}}+\left(D_{5\text{-}\mathrm{FU}\text{-}\beta \mathrm{CD}}\right)\right)+\left(D_{5\text{-}\mathrm{FU}}-\left(D_{5\text{-}\mathrm{FU}\text{-}\beta \mathrm{CD}}\right)\right)\left[1-K\left(c_{2}-c_{1}\right)\right]R\right\}$$

(11) $$D_{12}=\frac{1}{2}\left\{\left(\left(D_{5\text{-}\mathrm{FU}\text{-}\beta \mathrm{CD}}\right)-D_{5\text{-}\mathrm{FU}}\right)+\left(D_{5\text{-}\mathrm{FU}}-\left(D_{5\text{-}\mathrm{FU}\text{-}\beta \mathrm{CD}}\right)\right)\left[1-K\left(c_{2}-c_{1}\right)\right]R\right\}$$

(12) $$\;D_{21}=\frac{1}{2}\left\{\left(\left(D_{5\text{-}\mathrm{FU}\text{-}\beta \mathrm{CD}}\right)-D_{\beta \mathrm{CD}}\right)+\left(D_{\beta \mathrm{CD}}-\left(D_{5\text{-}\mathrm{FU}\text{-}\beta \mathrm{CD}}\right)\right)\left[1-K\left(c_{2}-c_{1}\right)\right]R\right\}$$

(13) $$D_{22}=\frac{1}{2}\left\{\left(D_{\beta \mathrm{CD}}+\left(D_{5\text{-}\mathrm{FU}\text{-}\beta \mathrm{CD}}\right)\right)+\left(D_{\beta \mathrm{CD}}-\left(D_{5\text{-}\mathrm{FU}\text{-}\beta \mathrm{CD}}\right)\right)\left[1-K\left(c_{2}-c_{1}\right)\right]R\right\}$$

where

(14) $$R={\left\{{\left[1+K\left(c_{2}-c_{1}\right)\right]}^{2}+4Kc_{1}\right\}}^{-1/2}$$

The calculated values for the limiting diffusion coefficients of these species (5-FU, *β*-CD, and 5-FU-*β*-CD) are indicated in Table 3.

The diffusion coefficients of free 5-FU (*D*_5-FU_ = 1.050 × 10^−9^ m^2^ s^−1^) and free *β*-cyclodextrin (*D_β_*_-CD_ = 0.399 × 10^−9^ m^2^ s^−1^) were obtained from *D*_11_ at X_1_ = 1 and from *D*_22_ at X_1_ = 0, respectively. The diffusion coefficient of the complex formed by these components (*D*_(5-FU-*β*-CD)_ = 0.390 × 10^−9^ m^2^ s^−1^) was estimated using Equation (15).

(15) $$R={\left\{{\left[1+K\left(c_{2}-c_{1}\right)\right]}^{2}+4Kc_{1}\right\}}^{-1/2}$$

This equation was derived from the Stokes–Einstein approximation [42], which relates the diffusion coefficient of a given species to its effective radius, *R*_h_, and, consequently, to its molecular volume.

When applying the theoretical Equations (10)–(13), the best agreement obtained between these predicted values and our experimental data (Table 2) was found when the binding constant *K* equal to (10.0 (±0.5) mol^−1^ dm^3^) was used. As a low value of *K*, it led us to conclude that the interaction between *β*-CD and 5-FU was weak. An identical situation was found for similar substances such as L-dopa [25] and paracetamol [47].

Supporting this fact was the low fraction of 5-FU species solubilized (or encapsulated) by the *β*-CD, *s*, estimated using Equation (16), and using the value of the diffusion coefficient of 5-FU at infinitesimal concentration, (*D*^0^_5-FU_ = 1.168 × 10^−9^ m^2^ s^−1^ [48]), and the values of the tracer diffusion coefficient of 5-FU in 0.007 and 0.010 of (mol dm^−3^) *β*-CD solutions; that is, (*D*_11_ = 1.011 × 10^−9^ m^2^ s^−1^) and (*D*_11_ = 1.023 × 10^−9^ m^2^ s^−1^), respectively.

(16) $$D_{11}=(1-s)\;D_{5\text{-}\mathrm{FU}}+sD_{\left(5\text{-}\mathrm{FU}\text{-}\beta \mathrm{CD}\right)}\;\approx \;(1-s)\;D_{5-\mathrm{FU}}^{0}+sD_{\left(5\text{-}\mathrm{FU}\text{-}\beta \mathrm{CD}\right)}$$

It was noted that the difference between the average of the tracer diffusion coefficient was approximately 13% of the limiting value ($D_{5\text{-}\mathrm{FU}}^{0}\;$= 1.168 × 10^−9^ m^2^ s^−1^ [48]). These deviations suggested that there was a solubilization of 5-FU in the more slowly diffusing complex of approximately 10% of 5-FU species solubilized by the *β*-CD (Table 4).

### 3.2. Ternary Mutual Diffusion Coefficients of Aqueous 5-FU (C_1_) + SDS (C_2_) Solutions

Table 5 shows the experimental ternary diffusion coefficients (*D*_11_, *D*_12_, *D*_21_, *D*_22_) of aqueous 5-FU (Component 1) plus SDS solutions (Component 2). Knowing that the critical micelle concentration for binary aqueous sodium dodecyl sulfate solutions (CMC) is 0.0083 (mol dm^−3^) [49], this study was carried out while considering the compositions of SDS before and after the micelle (CMC).

Based on the data in Table 5, it was verified that while for solutions at *C*_2_ < CMC, the *D*_11_ values were very close to the binary diffusion coefficients for 5-FU at the same compositions [48], for *C*_2_ > CMC, the *D*_11_ values found were lower, with deviations up to 17%. In relation to *D*_22_ values, it was observed that those obtained below the CMC (where the ionic dissociation of SDS was complete) were similar to the binary diffusion coefficients of aqueous SDS [49], and higher than those obtained above the CMC, where the micelles were formed and, consequently, their diffusion had to be hindered [50].

On the other hand, similar to what was observed in the 5-FU + *β*-CD system, the cross-diffusion coefficients *D*_12_ and *D*_21_ generally showed negative values, which meant that a coupled counter-current flow of 5-FU took place in this system in the opposite direction to the spontaneous flow of SDS and, consequently, that complexes were formed between 5-FU and SDS.

Support for the coupled diffusion was also given by the values of the *D*_21_/*D*_11_ ratio, which showed that one mole of diffusing 5-FU counter-transported up to 0.11 mol of SDS, whereas the *D*_12_/*D*_22_ values showed that one mole of diffusing SDS could counter-transport up to 0.21 mol of 5-FU; in both cases, in the range of concentrations above the CMC.

In the limit (X_1_ → 0), since the solution did not contain 5-FU, there could not be a gradient of this component that drove the SDS coupled flows. Under these circumstances, *D*_11_ represented the tracer diffusion coefficient of 5-FU in SDS aqueous solutions, and *D*_22_ the binary mutual diffusion coefficient of SDS. At the other limit, when X_1_ → 1, there was no concentration gradient of SDS, and therefore, it could not drive 5-FU coupled flows. In this case, *D*_11_ was the binary mutual diffusion coefficient of 5-FU, and *D*_22_ was the tracer diffusion coefficient of SDS in 5-FU aqueous solutions.

Taking into account that the tracer diffusion coefficients of 5-FU in 0.020 and 0.050 (mol dm^−3^) SDS solutions (*D*_11_ = 1.001 × 10^−9^ m^2^ s^−1^ and *D*_11_ = 0.965 × 10^−9^ m^2^ s^−1^, respectively) differed significantly from the diffusion coefficient of 5-FU at an infinitesimal concentration (*D*^0^_5-FU_ = 1.168 × 10^−9^ m^2^ s^−1^) value [48] (14% and 17%), it is possible to suggest that there was solubilization (encapsulation) of 5-FU molecules in the more slowly-diffusing micelles (*D*_micelle_ = 0.10 × 10^−9^ m^2^ s^−1^ [50]) and, consequently, to assume that these micelles included 5-FU molecules.

Considering the complexation equilibrium between 5-FU and SDS, and taking the approach that the diffusion coefficient value of the complex (5FU-SDS) was equal to that of the diffusion coefficient of the micelle (*D*_micelle_ = 0.10 × 10^−9^ m^2^ s^−1^ [50]), values for the limiting diffusion coefficients of the species present in this 5-FU + SDS system; i.e., for free 5-FU and free SDS and for the complex 5-FU-SDS, could be estimated. These values are shown in Table 6.

The use of the theoretical Equations (10)–(13), adapted for this 5-FU + SDS system under study, allowed us to find the relationship between the experimental mutual diffusion coefficients (Table 5) and the estimated values given in Table 6. The best agreement was obtained for a value of the association constant, *K*, equal to (20.0 (±0.5) mol^−1^ dm^3^) (a value of this binding constant that was double that found for the 5-FU + *β*-CD system)

Using Equation (17), the fraction of 5-FU species solubilized by the SDS micelles, *s*, in aqueous solutions of 5-FU plus SDS can be calculated:

(17) $$D_{11}=(1-s)\;D_{5\text{-}\mathrm{FU}}+sD_{\left(5\text{-}\mathrm{FU}\text{-}\mathrm{SDS}\right)}\;\approx \;(1-s)\;D_{5\text{-}\mathrm{FU}}^{0}+sD_{\left(5\text{-}\mathrm{FU}\text{-}\mathrm{SDS}\right)}$$

These values are collected in Table 7.

As could be ascertained, the *s* values were different from zero (*s* = 0.2), so we can say that solubilization (encapsulation) of 5 FU in this potential carrier was not negligible (between 16 and 19% of the 5-FU species was solubilized by the SDS), verifying that it was even more significant than that obtained with the other carrier, *β*-CD (*s* = 0.1)

## 4. Conclusions

We measured ternary diffusion coefficients of aqueous systems for the systems {5-fluorouracil + *β*-cyclodextrin (*β*-CD}) and {5-fluorouracil + sodium dodecyl sulphate (SDS)} at tracer and finite concentrations and at 298.15 K, with the objective of removing this polluting drug (5-fluorouracil) from the residual systems in which it is present. Relative to the last system, we investigated the behavior of these transport coefficients below and above the critical micelle concentration (CMC). Based on the negative cross-diffusion coefficients obtained for the two aqueous systems studied, we concluded that the interactions between 5-FU and both the *β*-CD and SDS molecules were not negligible, indicating the formation of complex species between 5-FU and both *β*-CD and SDC (that is, 5- FU was encapsulated by both carriers). However, SDS concentration gradients produced more significant counter-current coupled flows of 5-FU, promoting greater solubilization of the drug or, equivalently, a more efficient encapsulation of this drug; consequently, its use in removing the drug 5-FU will be more effective and lead to better results.

## Figures and Tables

**Table 1:** Sample descriptions.

Chemical Name	Source	CAS Number	Mass Fraction Purity ^1^
5-Fluorouracil	Sigma-Aldrich	54-21-7	>0.99
Sodium dodecyl sulfate	Merck	7732-18-5	>0.99
*β*-cyclodextrin	Sigma, Kawasaki, Japan (water mass fraction 0.131)	7585-39-9	≥0.97
Water	Millipore-Q water (18.2 MΩ·cm at 298.15 K)	7732-18-5	

^1^ As stated by the supplier.

**Table 2:** Experimental ternary diffusion coefficients (*D*_11_, *D*_12_, *D*_21_, *D*_22_) of 5-FU (*C*_1_) + *β*-CD (*C*_2_) aqueous solutions at *T* = 298.15 K and *P* = 101.3 kPa.

*C*_1_^1^	*C*_2_^1^	*X*_1_^2^	*D*_11_ ± S_D_^3^	*D*_12_ ± S_D_^3^	*D*_21_ ± S_D_^3^	*D*_22_ ± S_D_^3^
0.000	0.007	0.000	1.011 ± 0.020	0.013 ± 0.085	−0.030 ± 0.019	0.399 ± 0.012
0.0035	0.0035	0.500	1.008 ± 0.010	0.030 ± 0.085	−0.020 ± 0.029	0.405 ± 0.010
0.007	0.000	1.000	1.010 ± 0.020	−0.090 ± 0.015	−0.008 ± 0.019	0.427 ± 0.012
0.000	0.010	0.000	1.023 ± 0.002	0.040 ± 0.030	−0.020 ± 0.010	0.398 ± 0.010
0.012	0.008	0.375	1.015 ± 0.010	0.007 ± 0.001	−0.025 ± 0.009	0.431 ± 0.001
0.018	0.002	0.900	1.055 ± 0.020	−0.003 ± 0.001	−0.020 ± 0.046	0.462 ± 0.001
0.020	0.000	1.000	1.050 ± 0.029	−0.045 ± 0.007	−0.012 ± 0.010	0.465 ± 0.007

^1^ Concentrations in units of (mol dm^−3^). ^2^ *X*_1_ represents the 5-FU solute mole fraction. ^3^ Diffusion coefficients and standard deviation, *S*_D_, in units of (10^−9^ m^2^ s^−1^).

**Table 3:** Limiting diffusion coefficients, *D*_s_, of species present in the system 5-FU + *β*-CD at *T* = 298.15 K.

Species	*D*_S_/(10^−9^ m^2^ s^−1^)
5-FU	1.050 ^1^
*β*-CD	0.399 ^1^
5-FU-*β*-CD	0.390 ^2^

^1^ Estimated from *D*_11_ at *X*_1_ = 1 and from *D*_22_ at *X*_1_ = 0, respectively. ^2^ Estimated using Equation (15).

**Table 4:** Fraction of 5-FU species solubilized by the *β*-CD, s, in aqueous 5-FU (*C*_1_) + *β*-CD (*C*_2_) solutions at *T* = 298.15 K and *P* = 101.3 kPa.

*C*_1_^1^	*C*_2_^1^	*X*_1_	*s* ^2^
0.000	0.007	0.000	0.107
0.000	0.010	0.000	0.092

^1^ Concentrations in units of (mol dm^−3^). ^2^ *s* represents the fraction of 5-FU solubilized by the *β*-CD and that could be estimated from diffusion data using Equation (16), using (*D*_11_= 1.011 × 10^−9^ m^2^ s^−1^) and (*D*_11_ = 1.023 × 10^−9^ m^2^ s^−1^) for the tracer diffusion coefficient of 5-FU in 0.007 and 0.010 of (mol dm^−3^) *β*-CD solutions (*D*^0^_5-FU_ = 1.168 × 10^−9^ m^2^ s^−1^ [48] and *D*_(__5-FU-*β*-CD)_ = 0.390 × 10^−9^ m^2^ s^−1^).

**Table 5:** Experimental ternary diffusion coefficients (*D*_11_, *D*_12_, *D*_21_, *D*_22_) of 5-FU (*C*_1_) + SDS (*C*_2_) aqueous solutions at *T* = 298.15 K and *P* = 101.3 kPa.

*C*_1_^1^	*C*_2_^1^	*X*_1_^2^	*D*_11 ±_ S_D_^3^	*D*_12 ±_ S_D_^3^	*D*_21 ±_ S_D_^3^	*D*_22 ±_ S_D_^3^
				*C*_2_ < CMC ^4^		
0.000	0.004	0.000	1.160 ± 0.020	0.050 ± 0.015	−0.030 ± 0.069	0.789 ± 0.012
0.004	0.000	1.000	1.085 ± 0.020	0.010 ± 0.015	−0.008 ± 0.019	0.830 ± 0.012
0.018	0.002	0.375	1.090 ± 0.020	−0.016 ± 0.015	−0.040 ± 0.019	0.693 ± 0.012
0.010	0.000	1.000	1.051 ± 0.004	−0.012 ± 0.015	−0.002 ± 0.019	0.870 ± 0.012
				C_2_ > CMC ^4^		
0.000	0.020	0.000	1.001 ± 0.010	0.010 ± 0.014	−0.050 ± 0.009	0.378 ± 0.003
0.011	0.009	0.550	1.005 ± 0.010	−0.078 ± 0.014	−0.028 ± 0.009	0.367 ± 0.003
0.000	0.050	0.000	0.965 ± 0.012	0.025 ± 0.018	−0.101 ± 0.010	0.504 ± 0.001

^1^ Concentrations in units of (mol dm^−3^). ^2^ *X*_1_ represents the 5-FU solute mole fraction. ^3^ Diffusion coefficients and standard deviation, *S*_D_, in units of (10^−9^ m^2^ s^−1^). ^4^ CMC = 0.0083 (mol dm^−3^) [49].

**Table 6:** Limiting diffusion coefficients, *D*_s_, of species present in the system 5-FU + SDS at *T* = 298.15 K and *P* = 101.3 kPa.

Species	*D*_S_/(10^−9^ m^2^ s^−1^)
5-FU	1.050 ^1^
SDS	0.378 ^1^
5-FU-SDS	0.100 ^2^

^1^ Estimated from *D*_11_ at *X*_1_ = 1 and from *D*_22_ at *X*_1_ = 0, respectively. ^2^ By considering this diffusion coefficient of the complex (5-FU-SDS) equal to the diffusion coefficient of the micelle [50].

**Table 7:** Fraction of 5-FU species solubilized by the SDS micelles, *s*, in 5-FU (*C*_1_) + SDS (*C*_2_) aqueous solutions at *T* = 298.15 K and *P* = 101.3 kPa.

*C*_1_^1^	*C*_2_^1^	*X*_1_	*s* ^2^
0.000	0.020	0.000	0.156
0.000	0.050	0.000	0.190

^1^ Concentrations in units of (mol dm^−3^). ^2^ *s* represents the fraction of 5-FU solubilized (or encapsulated) by the SDS micelles, and can be estimated from diffusion data using Equation (17).

## Data Availability

Not applicable.
